# Cross-Talk Between Alveolar Macrophages and Lung Epithelial Cells is Essential to Maintain Lung Homeostasis

**DOI:** 10.3389/fimmu.2020.583042

**Published:** 2020-10-15

**Authors:** Elyse Y. Bissonnette, Jean-François Lauzon-Joset, Jason S. Debley, Steven F. Ziegler

**Affiliations:** ^1^ Centre de Recherche de l’Institut Universitaire de Cardiologie et de Pneumologie de Québec, Department of Medicine, Université Laval, Quebec City, QC, Canada; ^2^ Center for Immunity and Immunotherapies, Seattle Children’s Research Institute, Seattle, WA, United States; ^3^ Department of Immunology, Benaroya Research Institute, University of Washington School of Medicine, Seattle, WA, United States

**Keywords:** alveolar macrophages, airway epithelial cells, cytokines, extracellular vesicles, inflammation, macrophage heterogeneity

## Abstract

The main function of the lung is to perform gas exchange while maintaining lung homeostasis despite environmental pathogenic and non-pathogenic elements contained in inhaled air. Resident cells must keep lung homeostasis and eliminate pathogens by inducing protective immune response and silently remove innocuous particles. Which lung cell type is crucial for this function is still subject to debate, with reports favoring either alveolar macrophages (AMs) or lung epithelial cells (ECs) including airway and alveolar ECs. AMs are the main immune cells in the lung in steady-state and their function is mainly to dampen inflammatory responses. In addition, they phagocytose inhaled particles and apoptotic cells and can initiate and resolve inflammatory responses to pathogens. Although AMs release a plethora of mediators that modulate immune responses, ECs also play an essential role as they are more than just a physical barrier. They produce anti-microbial peptides and can secrete a variety of mediators that can modulate immune responses and AM functions. Furthermore, ECs can maintain AMs in a quiescent state by expressing anti-inflammatory membrane proteins such as CD200. Thus, AMs and ECs are both very important to maintain lung homeostasis and have to coordinate their action to protect the organism against infection. Thus, AMs and lung ECs communicate with each other using different mechanisms including mediators, membrane glycoproteins and their receptors, gap junction channels, and extracellular vesicles. This review will revisit characteristics and functions of AMs and lung ECs as well as different communication mechanisms these cells utilize to maintain lung immune balance and response to pathogens. A better understanding of the cross-talk between AMs and lung ECs may help develop new therapeutic strategies for lung pathogenesis.

## Introduction

The main function of the lung is to perform gas exchange while maintaining lung homeostasis despite environmental pathogenic and non-pathogenic elements contained in inhaled air. Considering that the volume of air inhaled every day is 5 to 8 L a minute, the prevalence of inflammation and pulmonary diseases is surprisingly low. Resident lung cells must discriminate between innocuous and harmful particles without creating unnecessary inflammation against inoffensive particles, while initiating an immune response against pathogens when necessary. Inappropriate or imbalanced immune response may underpin respiratory diseases. To maintain a proper balance, the lung needs specialized cells that can efficiently initiate and resolve inflammatory responses. Alveolar macrophages (AMs) and lung epithelial cells (ECs) are described in the literature as being the most important cells in the maintenance of lung homeostasis. AMs are the main immune cell type in the lung that determine the orientation and the magnitude of the immune response (1). In addition, they eliminate pathogens, apoptotic cells, and debris. On the other hand, a great number of publications claim that lung ECs are the main cell type keeping lung homeostasis with their antimicrobial activities acting as a barrier and a sensor of lung environment content (2). In reality, both AMs and ECs are the gatekeepers of the lung as they together are the first line of host defense and innate immunity. Furthermore, they communicate with each other to coordinate their actions to preserve lung homeostasis and gas exchange (3). Thus, in this review, we will briefly delineate the role of AMs and ECs and then focus on the everlasting cross-talk between AMs and lung ECs to maintain lung immune balance.

## AMs

### Origin and Heterogeneity

There are two types of macrophages found in the lung and named according to their location. AMs are found in the alveoli and airways and are easy to collect by bronchoalveolar lavage making them a highly studied cell type (4). There are also macrophages located in the alveolar septa and in the vascular adventitia that can be isolated from digested lung (5). These two macrophage populations can be distinguished using autofluorescence and surface markers (6). For the purpose of this review, we will focus on AMs that are found throughout the respiratory tract and are intimately associated with epithelial surfaces of both terminal airspaces and conducting airways (7).

For many years it was believed that AMs came from the differentiation of monocytes in the lung. Although this is true for some AMs (8), it is now well established that AMs mainly derive from embryo yolk sac and fetal liver cells (9–11). AMs are long-lived cells and a subpopulation of them can proliferate *in situ* to replenish themselves with a turnover rate of around 40% in one year (8, 12–14). This *in situ* proliferation required granulocyte-macrophage colony-stimulating factor (GM-CSF) and is controlled by mechanistic target of rapamycin complex 1 (mTORC1), a regulator of cell growth and proliferation (6, 15). Interestingly, GM-CSF also increases the expression of anti-apoptotic genes leading to long-lived cells (6). This maintenance of AM number is observed during homeostasis and under stress condition, but during acute inflammatory responses more macrophages are needed. Thus, there is rapid recruitment of monocyte-derived macrophages to the lung to eliminate pathogens (8, 16). These recruited macrophages promote lung inflammation whereas resident AMs dampen it (14, 17). During the resolution of inflammation, the majority of monocyte-derived macrophages undergo programmed cell death, while resident AMs survive and persist after the resolution phase (8). However, some recruited macrophages become phenotypically and functionally similar to resident AMs two months after infection showing that a part of AM population can also be replenished by monocyte-derived macrophages (18).

**Figure 1:**
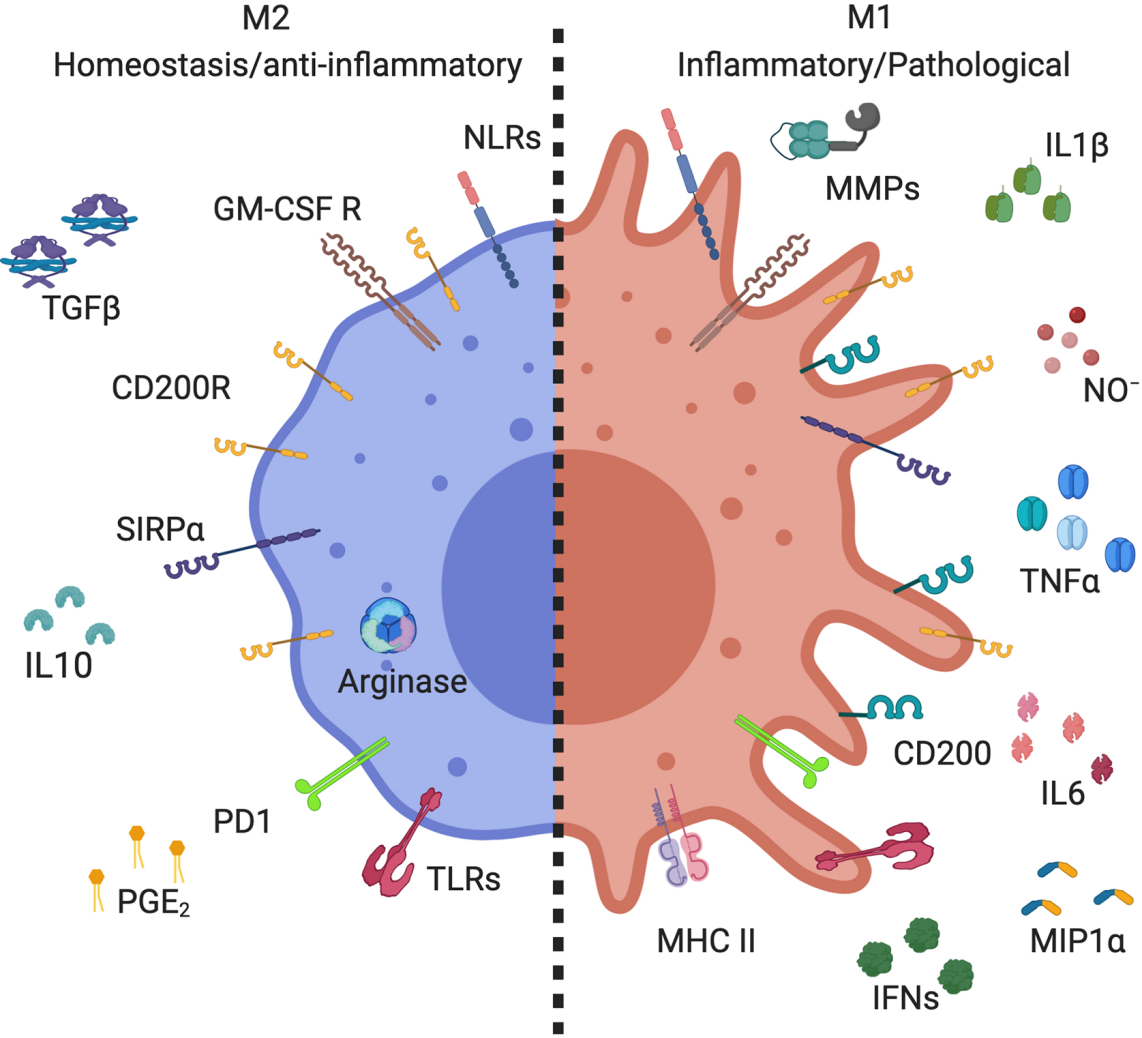
Markers expressed by alveolar macrophage subsets. Alveolar macrophages (AMs) expressed high levels of CD200R, SIRPα, GM-GSF R, and PPRs, including TLRs and NLRs. M2 AMs (homeostasis/anti-inflammatory) have high expression of arginase, and secrete anti-inflammatory mediators, such as IL-10, PGE_2_, and TGFβ. M1 AMs (inflammatory/pathological) increase their expression of CD200 and MHC II, as well as producing inflammatory mediators, such as metallopeptidase (MMPs), NO^-^, IL-1β, IFNs, IL-6, TNFα, and MIP-1α. Created with BioRender.com.

AMs are characterized by high expression of GM-CSFR, CD200R, and SIRP1α (**Figure 1**). Like other tissue macrophages, AMs show functional heterogeneity and plasticity depending on the microenvironment (1, 19). Tissue macrophages were first divided similarly to T helper lymphocytes; M1 being classically activated macrophages and M2 alternatively activated macrophages (20). M1 macrophages express high levels of pro-inflammatory mediators, whereas M2 express anti-inflammatory and wound healing mediators (19, 21) (**Figure 1**). The characterization of these populations of macrophages mostly results from *in vitro* stimulation. Macrophages stimulated with lipopolysaccharide (LPS) or inflammatory cytokines, such as interferon-γ (IFN-γ), display M1 phenotype with high expression of inducible nitric oxide synthase (iNOS), interleukin-1β (IL-1β), and tumor necrosis factor α (TNFα) (**Figure 1**) (22–24). These cells also express high level of MHCII and CD200 (25–27) and have cytotoxic and antitumoral properties (28, 29). On the other side, M2 macrophages arise in response to Th2 cytokines, IL-4, and/or IL-13 (30, 31). These macrophages express high level of arginase and anti-inflammatory cytokines such as IL-10 and transforming growth factor β (TGFβ) (**Figure 1**). They are involved in wound healing, tissue repair, and fibrosis, but have poor anti-microbicidal activity (32, 33), including low nitric oxide production (NO), as high arginase expression is known to inhibit NO production (34). Further studies demonstrated the existence of different subpopulations of M2 macrophages (35). Benoit et al. suggested dividing M2 macrophage subpopulations according to exposure agents; M2a referring to macrophages exposed to IL-4/IL-13, M2b to immune complexes and toll like receptor (TLR) agonists, M2c to IL-10 and glucocorticoid hormones, and M2d to TLR agonists through adenosine receptor (33, 35, 36). Thus, the dichotomous classification of M1/M2 does not align perfectly with Th1/Th2 immune response as previously suggested (37). Although M1 and M2a,b,c,d macrophages are phenotypically and functionally distinct macrophages (38, 39), they lack specific surface markers [reviewed by Wang et al. (40)]. Indeed, macrophage subpopulation identification relies on the relative intensity of marker expression and not on induction of expression. Thus, best practice characterization of macrophage subpopulations should include several markers of each phenotype (19).

M1/M2 classification of macrophages *in vitro* is easily done by adding specific stimuli to differentiate them, but identification of macrophage subpopulations is more complicated *in vivo* where numerous cytokines are present, particularly in inflammatory diseases. In reality, there is a continuum of macrophage populations with various functions and phenotypes that can be shifted from one to the other phenotype depending on the microenvironment and according to macrophage plasticity (41). In steady-state lung, AMs are in an immunosuppressed state and their phenotype is tightly control by the lung microenvironment. They express high level of CD200 receptor (CD200R) which is associated with M2 phenotype (42, 43) and are involved in the downregulation of immune inflammation (44, 45). This may be important for tolerating innocuous inhaled agent. However, in humans, there is no consensus on AM phenotypes. Studies showed data ranging from 8% to 50% of AMs are M2 in human steady-state lung (46, 47). These discrepancies may be caused by the infection/inflammation history long-term impact on AM functions and phenotypes. Alternatively, it could suggest the presence of multiple AM phenotypes in healthy lung responding to their specific environment to perform a variety of functions (14).

### AM Functions and Immune Response

AMs are well known for their role in maintaining lung homeostasis by phagocytosing microbes, dead cells, and other airborne particles to prevent unnecessary inflammation (48–51). Although AMs are considered poor antigen presenting cells (52–54), they can transport antigens to the draining lymph nodes (55). However, in the lung, antigen presentation is mainly mediated by dendritic cells. Interestingly, AMs suppress dendritic cell function and migration in and out of the airways to avoid immune responses against innocuous particles (53, 56, 57). Furthermore, AMs are known to downregulate T cell-dependent immune response in the lung by inducing FoxP3 expression in T cells (58, 59). A defect in this function is observed in asthma patients demonstrating the importance of AMs in the initiation of tolerance (60, 61). Depletion of AMs potentiates allergic asthma development and the severity of influenza infection, showing the significant role of these cells to dampen immune responses (48, 50, 51).

In steady-state, AMs execute anti-inflammatory functions to avoid immunopathology and the development of specific immune responses to harmless antigens. They are also the source of anti-inflammatory mediators such as IL-10, TGFβ, and prostaglandin E_2_ (PGE_2_) (1, 62, 63), facilitating the resolution of inflammation (64, 65). However, AMs maintain their capacity to be activated by pathogens and other danger signals *via* immune recognition of pathogen-associated molecular patterns by pattern recognition receptors (PRRs) including TLR, NOD-like receptors (NLRs) and C-type lectin receptors to initiate innate and adaptive immune responses (66–68). AMs are a primary source of cytokines and chemokines initiating immune responses, including TNFα, NO, IL-1β, IL-6, IFNs, and macrophage inflammatory protein (MIP)-1α (69, 70) (**Figure 1**). However, over production of these mediators contributes to the pathogenesis of inflammatory lung diseases such as acute lung injury, asthma, and chronic obstructive pulmonary diseases (COPD) (71–73). Thus, a rigorous regulation of AM secretory mediators is required to maintain lung homeostasis.

In addition to their role in modulating the immune response, AMs maintain lung homeostasis, in part, by internalizing and catabolizing lung surfactant which is critical for lung biomechanic and immunity (74, 75). A deficiency in GM-CSF signaling can lead to the dysregulation of AM surfactant clearance and causes accumulation of proteins and phospholipids in airspaces leading to pulmonary alveolar proteinosis (76). Interestingly, transplantation of functional AMs reduces alveolar proteinosis supporting the assumption that AMs are essential for surfactant metabolism (77). However, AMs are not the only cells that control surfactant levels. Alveolar epithelial type 2 cells are also involved in production and active removal of surfactant demonstrating the collaboration between these two cell types to maintain lung homeostasis (78).

## Alveolar and Airway ECs

### Origin and Heterogeneity, and Characteristics

Airway epithelium represents a tight barrier separating the organism from the external environment. In the tracheobronchial airway, the epithelium is pseudostratified, ciliated, and contains secretory cells. In the small airway, the epithelium becomes more cuboidal with increased club cells (79) formerly named Clara cells, which should be avoided given the origin of the experimentation (80). The alveoli are composed of two distinct EC types, alveolar epithelial type I cells that are thin and cover around 95% of the internal surface of the lung, and alveolar epithelial type II cells that are cuboidal secreting cells located between type I cells (81). Alveolar type I cells are specialized in gas exchange and alveolar fluid regulation (82, 83), whereas type II cells have secretory functions and constitute the progenitor cells of the epithelium (84).

The predominant cell types constituting the bronchial airway epithelium include basal progenitor cells, ciliated cells, secretory club cells, and goblet cells (79, 85). However, rarer and more specialized airway EC types have recently been better characterized such as neuroendocrine cells, tuft-like cells, and ionocytes (85–88). Over the past several years newer methodologies including single cell RNA sequencing (scRNA-Seq) and lineage tracing using pulse-Seq have allowed for better characterization, identification of cell type markers, and improved understanding of the evolution of these cell types in the airway epithelium in *in vivo*, *ex vivo*, and *in vitro* model systems (86, 88). Basal cells (identified by expression of *P63* and *KRT5*) are the airway progenitors or stem cells that have the ability to differentiate and replenish all subtypes of cells of the airway epithelium (89). Goblet cells (expressing *MUC5B* and/or *MUC5AC*) secrete mucins (90, 91), and ciliated cells (identified by expression of *FOXJ1* and *AcTub*) (90, 92) serve the critical physiologic role of facilitating mucociliary transport by propelling the airway mucus gel layer that overlies airway surface liquid proximally in the airways. Secretory club cells (expressing *CCSP*, *SCGB1A1*, and *SCGB3A2*) serve a protective role by both metabolizing inhaled toxins using cytochrome P450 in their smooth endoplasmic reticulum and through secretion of glycosaminoglycans, uteroglobin, and a surfactant-like substance (90, 93). Tuft cells, originally described in the intestine as chemosensory cells that facilitate Th2 inflammation through their production of IL-25 and thymic stromal lymphopoietin (TSLP), have recently been identified as a rare airway EC type (identified by expression of *POU2F3*) (94). The role of pulmonary neuroendocrine cells (PNECs), present in bronchial airway epithelium (identified by expression of *SYP*, *CHGA*, *PGP9.5*, *ROBO2*, and *ENO2*), and their secretion of bioactive amines and peptides, remains poorly understood (95). The recently identified airway EC type ionocyte (co-expressing *FOXI1* and *CFTR*), although rare appears to be a major source of CFTR expression and function in the airway (86, 88). Recent elegant studies employing scRNA-Seq together with lineage tracing with pulse-Seq to track differentiation of airway ECs *in vivo* in mice, have described how airway basal cells can directly differentiate into club cells, tuft cells, PNECs, and ionocytes, whereas ciliated cells and goblet cells are derived secondarily from club cells (86). In the alveolar lung compartment where gas exchange occurs, the epithelium is squamous and consists of alveolar type I cells, identified through their expression of *HOPX*, *PDPN*, *AQP5*, and alveolar type II cells identified by their expression of *SPB*, *SPC*, and *HT2-280* (96, 97).

The effectiveness of the lung epithelial barrier arises from its capacity to elaborate apical tight junctions with underlying adherent junctions (98). These intercellular junctions establish cell polarity and provide a selective permeability barrier regulating the movement of ions and macromolecules between the apical and basolateral face of the epithelium (99). Several membrane proteins are involved in these tight junctions such as claudin family, occludin, zonula occludens, and junction adhesion molecules (100, 101). A disruption in the epithelial barrier or a dysfunction/dysregulation of junction proteins contributes to lung pathologies such as asthma, cystic fibrosis, COPD, and acute respiratory distress syndrome (102–108).

Another type of intercellular contact that enable intercellular communication and metabolites and signaling molecules exchange between ECs is the gap junctions (109). These junctions, formed by channel proteins called connexins, play a major role in cellular coordination of cell functions and ensure the integration of metabolic activity of attached cells. Lung cellular interactions also involve pannexin glycoproteins and connexin proteins unopposed to gap junctions that form hemichannels allowing paracrine cell-cell communication (110, 111). These various junctions between airway ECs are important for epithelium barrier and functions.

### Lung EC Model Systems

The current “gold-standard” for studying the airway epithelium *in vitro* or *ex vivo* using primary airway ECs is the air-liquid interface (ALI) model system. In this approach, airway ECs are seeded into collagen-coated permeable transwells, and when they become confluent apical media is removed and cultures are maintained in an ALI environment and epithelial differentiation medium (e.g. PneumaCult ALI™; Stemcell™) for at least 21 days, generating an organotypic differentiated airway epithelial culture that closely resembles the *in vivo* airway (97, 112, 113). This model can be used to study EC responses to environmental insults (114, 115) and viral infection (116, 117), and can be modified to support co-cultures of airway ECs with stromal and/or immune cells (118–121). Model systems that closely approximate the *in vivo* characteristics of alveolar ECs are less well developed. When primary cultures of pediatric alveolar lung ECs are cultured *in vitro* they expand as *KRT5* expressing basal-like cells, however, when cultured at an ALI they demonstrate increased expression of markers for airway, but not alveolar ECs (122). Recently progress has been made developing distal lung directed differentiation protocols that utilize lung progenitor cells cultured in a 3D organoid phase, with or without mesenchymal cells (97, 123, 124). Although some of these models do generate alveolar type II-like cells (123, 124), these cultures are grown in submerged culture conditions and therefore are suboptimal in that they do not yet model the *in vivo* ALI environment (97).

### Alveolar and Airway EC Functions

In addition to its role as a physical barrier, the epithelium is crucial for lung biomechanics (78, 125). Secretion of surfactant by alveolar epithelial type II cells is critical to stabilize the structure of alveoli by reducing surface tension at the air-liquid interface to avoid alveolar collapse (74). The absence or deficiency/inactivation of surfactant causes severe respiratory disorders such as neonatal respiratory distress syndrome and acute respiratory distress syndrome (126). On the other hand, impaired surfactant catabolism by AMs and/or alveolar epithelial type II cells leads to accumulation of surfactant in alveoli and is associated with lung proteinosis causing respiratory failure (127). Pulmonary surfactant is composed of around 90% lipids, mainly phospholipids (80%–85%) and some neutral lipids (5%–10%), and 8%–10% proteins, including two hydrophilic proteins, surfactant protein (SP)-A and SP-D, and two hydrophobic proteins, SP-B and SP-C. The proportion of all the constituents of pulmonary surfactant is important for the biomechanical functions of the film at the air-liquid interface reducing surface tension (125) and also for protecting against pathogens.

Protection against microbes entering the lung is crucial to maintain gas exchange. One line of defense of airway ECs is the production of a plethora of antimicrobial proteins and peptides such as β defensins, LL-37, lysozyme, lactoferrin, NO, and secretory leukocyte proteinase inhibitor (128, 129). The surfactant proteins, SP-A and SP-D, produce by type II alveolar ECs are also involved in pathogen clearance *via* several mechanisms including binding, agglutination, anti-microbial and fungal effects, and enhancement of neutrophil and AM phagocytosis and killing (130, 131). The production of antimicrobial peptides is stimulated by the activation of PRRs on lung ECs such as TLR. There are at least 10 TLRs expressed by lung ECs that recognise distinct pathogen-associated molecular patterns derived from viruses, bacteria, mycobacteria, fungi, and parasites (129, 132). Lung ECs express other PRRs such as the RIG-I-like receptor viral sensors RIG-1, MDA5, and LPG2, in addition to NLRs, C-type lectin, and inflammasome components (133–137) that participate to the immune responses.

### Modulation of Immune Response

Lung ECs contribute to local immune response through the production of a multitude of modulatory mediators. The stimulation of PRRs on lung ECs by viruses induces the production of type I and III IFNs which in turn induce expression of hundreds of interferon-stimulated genes (ISGs) (117), the protein products of some of which have local direct antiviral effects while others promote cellular immunity. Stimulation of lung EC PRRs also induce a number of pro-inflammatory cytokines and chemokines including IL-1β, IL-6, IL-8, TNF, GM-CSF, MIP-1α, RANTES, and monocyte chemoattractant protein (MCP)-1 (138–140). Together these ISGs and mediators promote the recruitment and activation of inflammatory cells to eliminate pathogens.

In addition to the production of inflammatory mediators, lung ECs contribute to the regulation of inflammation and airway remodeling through the production of anti-inflammatory products including IL-10, TGFβ, lipoxins, resolvins, protectins, and PGE_2_ (139, 141–143). Overall, the secretion of cytokines, chemokines, and lipid mediators by lung ECs help to shape a balanced immune response. However, EC aberrant secretion of pro-inflammatory cytokines lead to lung pathologies, as observed in pulmonary allergic inflammation where lung EC production of IL-25, IL-33, and TSLP are linked to the initiation and progression of the disease (116, 144, 145). Lung ECs also contribute to the pathogenesis of COPD through the production of a plethora of inflammatory mediators (146).

Lung ECs can also modulate immune cells through physical interaction *via* immunomodulatory surface molecules such as CD200, program death-ligand (PDL)-1, and CD47 (147–149). The binding of CD200 to its receptor, CD200R which is highly expressed on AMs, downregulate the secretion of inflammatory cytokines upon LPS stimulation (42), suggesting an immunomodulatory function of lung ECs. This regulation affects other immune cells such as T cells and dendritic cells that also express CD200R (150). Similarly, the binding of PDL-1 to its receptor PD-1 dampens AM secretion of inflammatory cytokines and negatively regulates T cell effector functions (151, 152), whereas binding of CD47 to SIRP1α expressing macrophages downregulate phagocytosis (153, 154). Thus, there is a breadth of evidence demonstrating the importance of lung ECs in regulating local immune responses, by interacting/communicating with immune cells, including AMs.

## Cross-Talk Between AMs AND LUNG ECs

**Figure 2:**
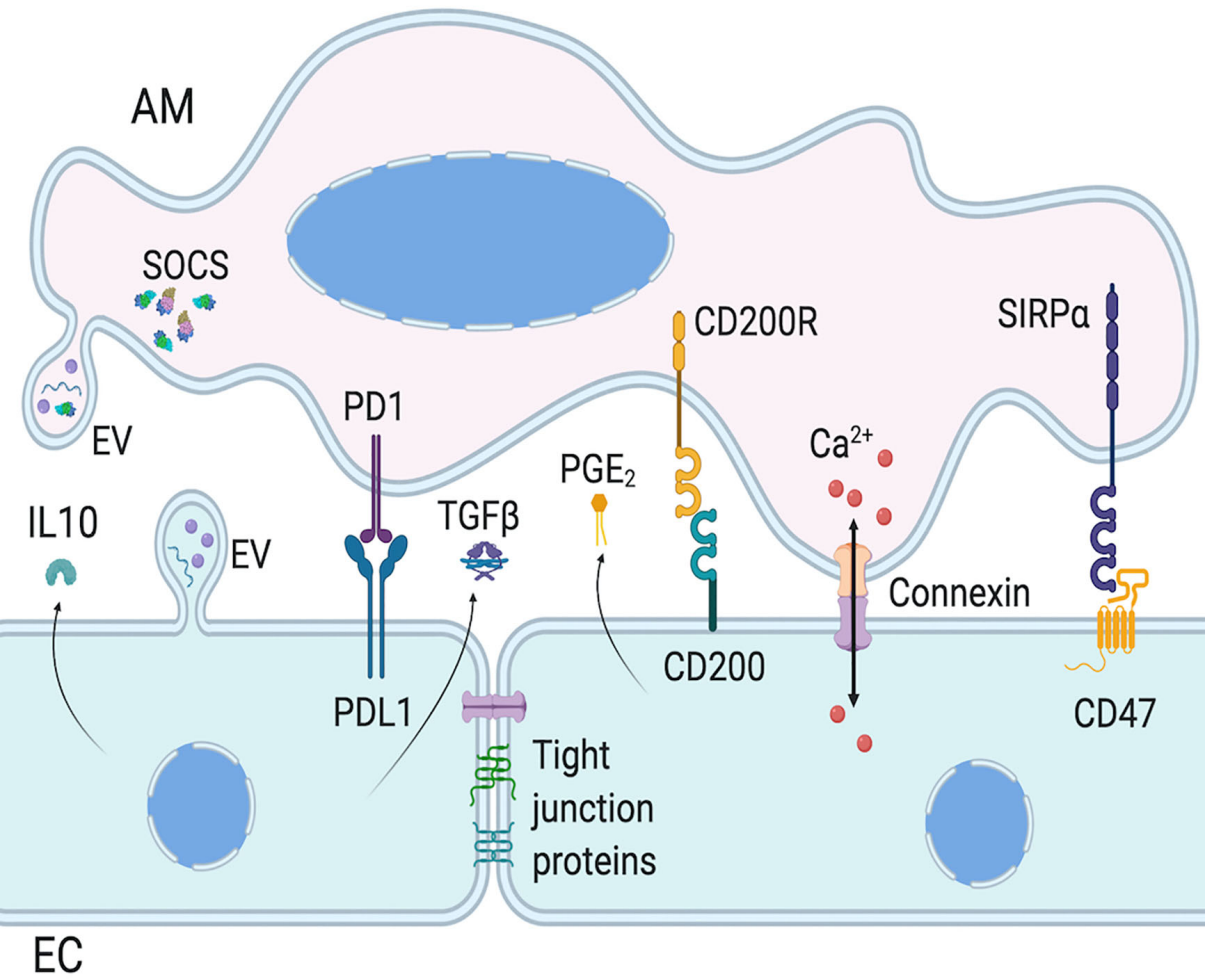
Intercellular communication between AMs and ECs. Epithelial cells (ECs) and alveolar macrophages (AMs) communication involves surface protein interaction, as well as mediator secretion and extracellular vesicles (EVs). AM activation is regulated by EC expression of PDL1, CD200, and CD47, which activate AM cognate receptor, respectively PD1, CD200R, and SIRPα. EC also regulate AM functions *via* paracrine secretion of mediators, such as IL-10 and PGE_2_. AM and EC functions can also be regulated with the release of EVs which can have inflammatory or anti-inflammatory functions, *via* EV surface proteins, cytokines or miRNAs. Of note, AMs constitutively secrete EVs containing SOCS. Finally, AMs and ECs can form gap junctions to allow bi-directional intercellular metabolic synchronicity, including Ca^2+^ waves. Created with BioRender.com.

Given that lung ECs and AMs are the first cell types being in contact with pathogens, they must coordinate their actions to eliminate pathogens without causing too much damage to the lung, either *via* direct cell-cell contact or secretion of molecules (**Figure 2**). In steady-state, ECs maintain AMs in a quiescent state. AMs adhere to the epithelium *via* extracellular membrane proteins such as CD200R, PD-1, and SIRP1α and their ligands on ECs, respectively CD200, PDL-1, and CD47 (42, 147, 149, 151, 155, 156). In addition to maintaining cells in close proximity to increase paracrine communication, these protein interactions downregulate AM activation. The contiguity of these two cell types may also allow the regulation of AMs by anti-inflammatory cytokines secreted by lung ECs such as IL-10 and TGFβ (139, 141). The loss of these regulatory interactions, either due to epithelial damage or sensing of pathogens in the airways, lead to the activation of AMs and initiation of inflammatory response. Whether the epithelium is the first to stimulate AM pro-inflammatory reaction is still a matter of debate, and may depend on the nature of the pathogen, but both of them are needed for adequate immune response.

### Alveolar Gap Junction Channel

Gap junctions are usually an intercellular contact between ECs, but they are also found between AMs and alveolar ECs. In mouse, a subset of AMs express connexin 43 enabling the formation of gap junctions with ECs (157). These gap junctions allow waves of Ca^2+^ signal to travel from AMs to ECs and vice-versa, as observed with LPS stimulation causing cyclic and synchronized calcium spikes in both cell types. Mice with macrophages deficient in connexin 43 had higher levels of pro-inflammatory cytokines in bronchoalveolar lavage that originated from both AMs and airway ECs (157). This supports the bi-directional anti-inflammatory role of AM-epithelium gap junction channel, and is essential to reduce LPS induced lung inflammation and injury. The presence of connexin 43 gap junctions between macrophages and ECs was also shown using human cells (158, 159), suggesting its involvement in human lung cell communication. However, the exact role of this communication is still undetermined.

### Extracellular Vesicles

Recently, extracellular vesicles (EVs) have emerged as a novel communication mechanism to exchange proteins, lipids, and genetic material between cells. These vesicles are divided into three categories, apoptotic bodies, exosomes, and microvesicles. The release of apoptotic bodies during apoptosis is well known and will not be discussed here [reviewed by Battistelli M et al. (160)]. Exosomes and microvesicles are released in steady-state conditions. The main difference between these two EVs resides in their size and origin. Exosomes are 40-120 nm in diameter and are released from endosomes fusing with the membrane, whereas microvesicles (also called microparticles or ectosomes) are 50–1,000 nm and are formed by the outward budding and fission of the plasma membrane (161, 162). Given the overlapping range of size and composition, and the difficulty of isolating exosomes and microvesicles separately to discriminate their specific functions (163), we will refer to EVs as an umbrella term for both exosomes and microvesicles in this review.

EVs are found in most human body fluids, including bronchoalveolar lavages (164). These EVs express membrane surface proteins, cytoskeletal and cytoplasmic proteins, cytokines, mRNAs, and miRNAs (**Table 1**). EVs may explain in part how cytokines/chemokines reach physiologic concentrations to affect target cells. In steady-state, EVs in bronchoalveolar lavages come largely from AMs and lung ECs (**Figure 2**), but during inflammatory response, infiltrating cell types also produce them (166–168, 171, 172, 174–177), as they are involved in immune responses, inflammation, bacterial and viral sequestration.

**Table 1:** Extracellular vesicle content.

Categories	Examples	References
Membrane surface proteins	CD3, CD14, CD40, CD54, CD63, CD80, CD81, CD86, MHCI, MHCII, tetraspanin, mucin, ion transport and ion channel proteins	Admyre et al. (164) Gupta et al. (165) Kulshreshtha et al. (166)
Cytoskeletal proteins	Tubulin, actin, moesin, radixin, ezrin	Gupta et al. (165) Kesimer et al. (167)
Cytoplasmic proteins	Heat chock proteins, mucin, annexin, cytokines, chemokines, complement C3, suppressor of cytokine signaling 1 (SOCS1), SOCS3	Kesimer et al. (167) Gupta et al. (165) Soni et al. (168) Bourdonnay et al. (169) Speth et al. (170)
Nucleic acids	mRNAs	Kesimer et al. (167) Lee et al. (171)
	miR-210, miR-320a, miR-221, miR-17, miR-3960, miR-1246, miR-4497	Fugita et al. (172) Lee et al. (171) Lee et al. (173) Gupta et al. (165)

EVs released by AMs and lung ECs can modulate each other in a pro- and anti-inflammatory manner. AMs constitutively secrete EVs containing suppressor of cytokine signaling (SOCS) proteins, which inhibit the inflammatory STAT pathway (**Figure 2**) (169). Lung ECs barely express SOCS proteins, however they secrete mediators, such as PGE_2_ and IL-10, that increase SOCS protein secretion by AMs. EVs containing SOCS proteins are taken up by lung ECs and downregulate cytokine-induced STAT activation, maintaining ECs in a quiescent state and limiting tumor transformation of normal ECs (170). Furthermore, vesicular SOCS can dampen allergic airway inflammation by inhibiting EC production of type 2 cytokines (178). Thus, EVs are crucial to maintain lung homeostasis through cell-cell communication, but they can also be involved in lung pathogenesis.

Indeed, content and concentration of EVs in bronchoalveolar lavage change during lung inflammation. However, there is still controversy on which cell types initiate immune responses. Under hyperoxia-induced oxidative stress, lung ECs secrete EVs to activate pro-inflammatory functions of AMs, facilitating the recruitment of immunomodulatory cells involved in lung injury (171, 176). In contrast, after pulmonary LPS exposure, AMs are the first producers of EVs containing inflammatory mediators which activate lung EC inflammatory response (168). In allergic inflamed lung, inhibition of EV secretion alleviates asthma features, although the cellular origin of EVs is unknown (166). Interestingly, intranasal transfer of EVs from bronchoalveolar lavage of tolerized mice prevent allergic sensitization, including production of IgE, Th2 cytokines, and lung inflammation (179). Yet, the functions of EVs produced by AMs and lung ECs are underexplored, and may have beneficial or detrimental effects depending on the context. More research is needed to better understand the communication between these two cell types in lung homeostasis and diseases.

## Conclusion

AMs close proximity to lung ECs allow them to communicate using gap junctions, surface membrane molecules, soluble mediators, and EVs. In steady-state, AMs and ECs downregulate each other to avoid unnecessary inflammation. However, each cell type can activate the other one to initiate an immune response when required.

There is a plethora of publications on AMs and lung ECs; yet, very few of them investigate their interactions under lung homeostatic conditions and how this interaction is altered in pathological conditions. Furthermore, numerous studies use macrophages derived from bone marrow or monocytes as surrogate for AMs, even though AMs are functionally and phenotypically different from other macrophages. Thus, to extend our knowledge on the interaction between AMs and ECs in lung steady-state and diseases, it is essential to perform *in vivo* experiments or to harvest AMs from bronchoalveolar lavage for *in vitro* co-culture with human primary lung ECs which show normal differentiation patterns (unlike epithelial cell lines) (180). In addition, *in vitro* co-culture systems of AMs and ECs should be performed at air-liquid surface, which mimics more closely the *in vivo* situation, including the formation of functional tight junctions (181). This more representative model may help improving our knowledge on AMs and lung ECs communication and their collaboration in the maintenance of lung homeostasis and response to pathogens and injuries. A better understanding of AMs and lung ECs cross-talk may help develop new therapeutic strategies for lung pathogenesis.

## Author Contributions

EB wrote the core of the manuscript, J-FL-J and JD made substantial intellectual contribution, J-FL-J drew the figures, and SZ reviewed the manuscript. All authors contributed to the article and approved the submitted version.

## Funding

EB is funded by Canadian Institutes of Health Research (grant PJT-152876) and Quebec Respiratory Research Health Network. J-FL-J is funded by Quebec Respiratory Research Health Network. JD is funded by the National Institutes of Health (grants U19AI125378, K24AI150991, and R01HL128361), and SZ is funded by the National Institutes of Health (grant U19AI125378).

## Conflict of Interest

The authors declare that the research was conducted in the absence of any commercial or financial relationships that could be construed as a potential conflict of interest.
